# Thermoplastic Pultrusion Process of Polypropylene/Glass Tapes

**DOI:** 10.3390/polym15102374

**Published:** 2023-05-19

**Authors:** Fausto Tucci, Felice Rubino, Germana Pasquino, Pierpaolo Carlone

**Affiliations:** 1 Department of Industrial Engineering, University of Salerno, 84084 Fisciano, Italy; pcarlone@unisa.it; 2 Department of Civil and Mechanical Engineering, University of Cassino and Southern Lazio, 03043 Cassino, Italy; frubino@unisa.it; 3 Universitas Mercatorum, 00186 Rome, Italy

**Keywords:** pultrusion, thermoplastic, polypropylene, glass, composite, pre-impregnated, tapes, testing

## Abstract

The present work focuses on the pultrusion of pre-impregnated glass-reinforced polypropylene tapes. An appositely designed laboratory-scale pultrusion line, consisting of a heating/forming die and a cooling die, was used. The temperature of the advancing materials and the pulling force resistance were measured by using thermocouples embedded in the pre-preg tapes and a load cell. From the analysis of the experimental outcomes, we gained insight into the nature of the material–machinery interaction and the transitions of the polypropylene matrix. The cross-section of the pultruded part was analyzed by microscope observation to evaluate the distribution of the reinforcement inside the profile and the presence of internal defects. Three-point bending and tensile testing were conducted to assess the mechanical properties of the thermoplastic composite. The pultruded product showed good quality, with an average fiber volume fraction of 23% and a limited presence of internal defects. A non-homogenous distribution of fibers in the cross-section of the profile was observed, probably due to the low number of tapes used in the present experimentation and their limited compaction. A tensile modulus and a flexural modulus of 21.5 GPa and 15.0 GPa, respectively, were measured.

## 1. Introduction

Sustainability is one of the most relevant drivers in the current activities of research and industry. The paradigms of sustainability are accounted for and applied throughout the whole life of a product: harvesting of the raw material, design, manufacturing, transportation, installation, in-service life, maintenance, and end of life [1]. Thermosetting polymeric matrix composites are widely used in several sectors from high-end applications to mass-consumption products thanks to their low weight, high specific properties, easy manufacturability, and competitive production time and costs. However, the main drawbacks for the future employment of such materials derive from the impossibility of recycling the thermoset systems and recovering the matrix and reinforcement phases. Therefore, the only way to reuse the thermosetting composites consists of grinding the composite wastes and using the fragments as particle reinforcement in concrete structures. This places these materials at the borders of the sustainability paradigm.

For this reason, research and industry are pushed towards the improvement of the manufacturability of thermoplastics and to increase the diffusion of thermoplastic matrix composites in the markets [2]. Low-viscosity polymers and off-line combinations of matrices and reinforcements are currently explored as suitable strategies to improve the processability of thermoplastic composites, and remarkable improvements have been accomplished in recent years [3]. Among them, the off-line combination of matrix and fibers is still the most commonly adopted strategy [4]. The off-line impregnation involves the use of pre-impregnated fabrics, tissues, or tapes with precisely controlled compositions. This approach increases the costs of raw materials and storage, but, on the other hand, it simplifies the manufacturing process and generally increases the product quality in terms of reducing voids and inclusion contents, and improving surface appearance.

The pultrusion process has been adopted for decades to produce continuous fiber-reinforced thermosetting composites employed in several sectors, from aeronautics to automotive and civil engineering [5]. The significant advantages in terms of mechanical performance of the final product, productivity, automation, and low energy consumption are promoting the redesign of the conventional pultrusion line to manufacture thermoplastics-based composites. In the case of thermoplastic pultrusion, two process variants currently exist according to the raw materials adopted: injection pultrusion of low-viscosity reactive thermoplastics and dry fibers, or pultrusion of pre-impregnated roving [6,7]. The first technique, despite being easier to implement since it behaves like the conventional thermosetting pultrusion and the knowledge about the processing of thermosetting can be extended to thermoplastic systems, presents some complexities to ensure a proper impregnation of the fibers due to the higher viscosity of the latter polymers [8]. The second technique, on the other hand, represents an easier way to perform the thermoplastic pultrusion process and, additionally, it achieves better control of the reinforcement volume fraction [8]. The raw materials suitable for this technique can be classified into three distinct categories: (i) commingled yarns consisting of filaments of matrix and reinforcement entangled with each other; (ii) towpregs, where the intimate contact between fibers and matrix is achieved mechanically using the thermoplastic in fine powder form; and (iii) pre-preg tapes (also known as pre-consolidated tapes, PCTs), where unidirectional fibers are impregnated with molten polymer by using a procedure similar to the conventional pultrusion [4]. The first two classes represent a viable solution for thermoplastic pultrusion, especially if recycled polymers are used. They, indeed, can employ polymers coming directly from the recycling chain in the form of filaments or micrometer pellets without the need for secondary processing and, in addition, they are less constrained by the properties of the raw materials. For these reasons, these approaches have been investigated in the past decade and several contributions can be found in the literature dealing with distinct aspects of these processes, from the manufacturing strategy to the impregnation mechanism of the fibers and to the mechanical properties of pultruded products [9,10,11,12,13]. However, the use of commingled yarns and towpregs, despite their flexible and cost-effective processability, has some manufacturing complexities related to the achievement of an optimal impregnation of the fibers and avoiding the formation of voids and pores, which cause deterioration of the performance of the final products [13]. Pre-preg tapes, on the other hand, combine the advantage of easy production and scalability of the pultrusion process with better consolidation between the matrix and the reinforcement and a high alignment of the fiber, achieving well-designed parts [14,15,16]. To date, few studies have focused on pre-preg tapes. Most studies are mainly devoted to the manufacturing of raw materials and their properties, while the study of the pultrusion process has been a secondary aspect [4,17]. This highlights the need to achieve a better understanding of the physical phenomena involved in thermoplastic pultrusion, the interaction of the materials with the machinery, and the influence of the processing conditions on the mechanical performance of the pultruded products.

In pre-pregs pultrusion, the line consists of a heating/forming die with a converging geometry cavity, followed by a cooling die with a constant cross-section cavity. In the heating die, the thermal energy, provided by an external heating system, melts the polymeric phase and the convergent cavity compacts the tapes to obtain the designed cross-section shape and fiber volume fraction. In the cooling die, the heat is removed by a cooling circuit determining the solidification of the polymeric phase and the full consolidation of the pultruded when it reaches the exit of the line [8].

During the phase transformations of the polymeric system inside the converging section of the heating die, the nature of the interaction between the material and the die walls changes, showing distinct behaviors from bulk compaction to viscous drag and solid friction, individuating different zones along the pultrusion line. The bulk compaction is generated by the convergent geometry of the cavity and depends on the converging angle and the local pressure. The viscous drag occurs when the temperature of the polymer in contact with the die walls overcomes the melting point. The viscosity of the thermoplastic, the pulling speed, and the thickness of the liquid polymeric interlayer formed between fibers and die influence the intensity of the viscous forces. Finally, the solid friction establishes at the final portion of the cooling die due to the sliding contact between the solidified composite and the cavity walls [18]. The resistance to the pulling force exerted by the processing material is the combination of these interactions and is the main factor responsible for residual stress in the polymeric matrix composite. The on-line evaluation of the pulling forces allows estimating the local interactions between processed material and die walls, providing insight into the internal transformations and the main phenomena occurring inside the die [19]. It allows the users to calibrate the process parameters and mitigate detrimental effects.

The present study aimed to investigate the pultrusion of glass-reinforced polypropylene pre-preg tapes. The combined analysis of forces and internal temperature provided a dataset to determine the phase transformations and their influence on the resistance to the pulling force. This kind of analysis, applied in the literature to study the thermoset matrix pultrusion [19], to the authors’ best knowledge, has never been documented before in scientific articles on the study of thermoplastic matrix pultrusion. The produced profiles were also analyzed by ultrasonic inspection and mechanical testing. The conducted analyses demonstrate the feasibility of the pultrusion process to realize composite profiles from pre-impregnated tapes. The study of the response to the pulling force is a valuable tool to deepen into the behaviors affecting the process and invites further investigations and developments.

## 2. Materials and Methods

The 6.35 mm wide commercial tapes produced by CompTape BV (Delft, The Netherlands) are composed of a roving in E-glass continuous filaments (Owens Corning SE4849, tex. number 2400) and polypropylene matrix (Moplen RP348U). Twenty-four tapes were arranged in six lines with four tapes each to realize a rectangular cross-section composite profile 25 mm wide and 4 mm thick, with a volume fraction of reinforcement of 23%.

**Figure 1:**
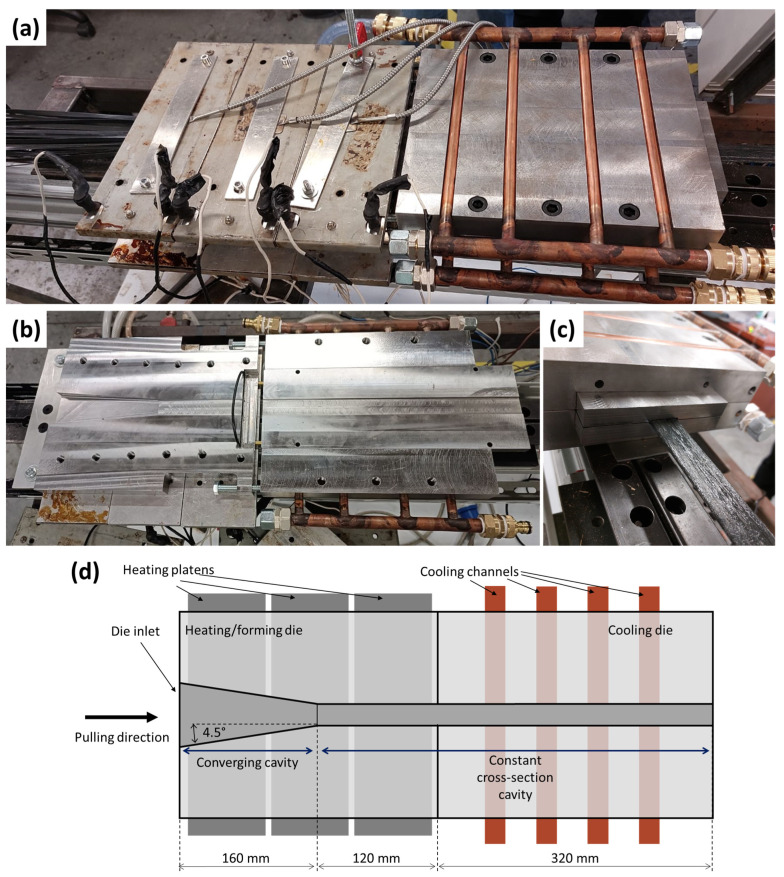
(**a**) Pultrusion setup while processing glass/polypropylene tapes; (**b**) view of the internal cavities of the dies; (**c**) obtained pultruded profile; (**d**) schematic of the heating/forming die and cooling die.

The laboratory-scale pultrusion line consists of the heating/forming die and the cooling die and is reported in Figure 1a. The features and the dimensions of the thermoplastic pultrusion dies realized for this experimental activity are reported in Figure 1d. The main difference between thermoplastic and thermoset pultrusion is in the dies. The conventional thermoset pultrusion die consists only of a heating/forming die [20], in which the polymerization reaction of the resin system is activated by thermal energy. In some cases, the die could present a converging entrance, but most of it has a constant cross-section [21]. The thermoplastic matrices need to be heated over the melting temperature, consolidated, and then cooled to achieve a solid composite profile. Therefore, the thermoplastic pultrusion lines are equipped with a heating die presenting a converging entrance to melt the matrix and consolidate the material, and a cooling die in charge of the absorption of the thermal energy to solidify the thermoplastic matrix and provide a gradual cooling of the pultruded composite.

Figure 1b,d shows the geometry of the cavities of both dies. The heating/forming die is 280 mm long. The thermal energy is provided by 3 couples of electrical heating plates monitored by J-type thermocouples and regulated by PID controllers to keep the temperature at 200 °C ± 5 °C. The die presents a converging geometry in the first 160 mm, where the cross-section gradually reaches the final shape of the profile. The following zone of the heating/forming die consists of a 120 mm long straight cavity. The cooling die is mechanically connected to the heating/forming die. The dies have been designed to achieve continuity between the two cavities. The heat is removed by a water-cooling system regulated by an industrial chiller with a temperature setpoint of 13 °C. A caterpillar pulling system provides the traction to move the materials along the entire line. The pulling speed was set at 170 mm/min.

During the test, the internal temperature of the composite was measured by adopting the traveling thermocouple method [22]. The thermocouple should be as non-invasive as possible in order to minimize the influence on the measured temperature. With this purpose, a K-type thermocouple having a bulb diameter of 0.2 mm (occupying about 0.03% of the cross-section) was adopted. The pulling force was monitored by using a compressive button load cell. The thermocouple and the load cell signals were acquired by using an Arduino board connected to a laptop. The fiber cutting method was applied to measure the local resistance to the pulling force [19]. This method consists of cutting off the tapes upstream of the entrance into the heating/forming die and acquiring the load measured by the load cell. The position of the cut section can be determined considering the pulling speed $v_{pull}$ and the cutting time $t_{c}$, as indicated in Equation (1):

(1) $$x_{c}\left(t\right)=v_{pull}\left(t-t_{c}\right)\;\;.$$

The total resistance force acquired by the load cell Q decreases while the cutting section advances within the dies, due to the continuous reduction in the interaction surface extension. Therefore, the local resistance $R_{L}$ can be evaluated as:

(2) $$R_{L}\left(x\right)=-\frac{dQ\left(x_{c}\right)}{dx}\;\;,$$

where $Q\;\left(x_{c}\right)$ represents the total load measured as a function of the cut section advancement.

The opposite of the derivative of the measured load corresponds to the local resistance. The analysis of the local resistance determines the end of the pultrusion test due to the cut-off of the feed tapes.

Several samples were collected from the pultruded composite for testing and subsequent analyses. The samples were picked from the composite profile produced in steady-state conditions (avoiding the transitory initial and final stages of the process) and where the thermocouple was not present.

**Figure 2:**
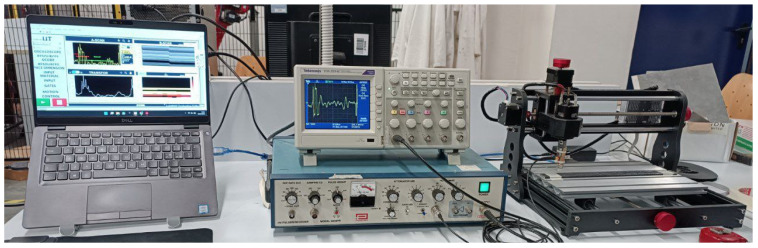
Ultrasonic inspection equipment.

Five random zones of the pultruded composite, 50 mm × 15 mm in size, were analyzed by ultrasonic inspection. The ultrasonic equipment consisted of a high-voltage pulser-receiver (Olympus Panametrics NDT 5058 PR, Huston, TX, USA) connected with a piezoelectric probe (Karl Deutsch S 12 PB 1–7, Wuppertal, Germany) designed for the inspection of thin composites. The probe was positioned and moved across the composite surface by using a bench-scale CNC (Computerized Numerical Control) system (Mostics 3018 Pro). Finally, the return signal was collected by an oscilloscope (Tektronix TDS2000C, Beaverton, OR, USA). The full equipment was controlled by a LabVIEW code, which coordinated the ultrasonic generator and the CNC moving system, and analyzed the data acquired by the oscilloscope. In particular, the code acquires the reflection of impulsively emitted signals and analyzes the reflected spectrum of each acquisition point to define a scan of the observed window. The acquisition system is depicted in Figure 2.

A sample was cut, embedded in mounting resin, and polished for macroscopic observation. The cross-section was analyzed by using a stereomicroscope (Motic SMZ171, Wetzlar, Germany).

**Figure 3:**
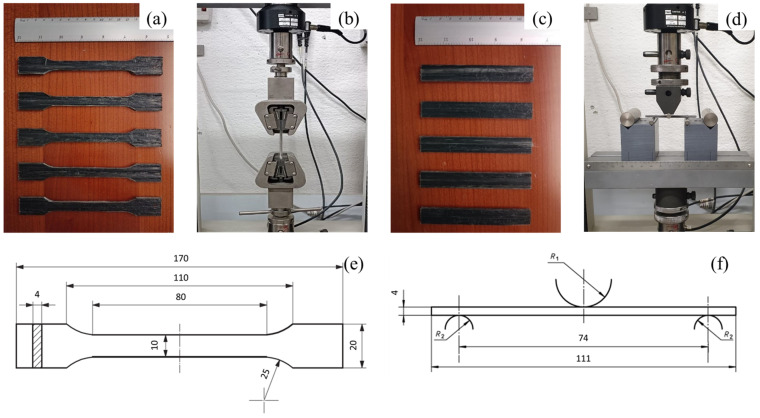
Samples (**a**), setup (**b**), and dimensions (**e**) for tensile testing; samples (**c**), setup (**d**), and dimensions (**f**) for three-point bending testing.

Ten samples were extracted from the pultruded composite. Five of them were shaped for tensile testing (Figure 3a) and the other five for three-point bending testing (Figure 3c), according to the prescriptions of ISO 20753 and ISO 14125 standards, respectively. An MTS Insight testing machine was used for the tensile and three-point bending testing. The tensile testing (Figure 3b) was conducted using a loading velocity of 10 mm/s, while the three-point bending testing (Figure 3d) was performed using a testing velocity of 5 mm/s.

## 3. Results and Discussion

Twenty-four pre-preg tapes were processed to produce a compact pultruded profile in glass-reinforced polypropylene. The tapes, entering the heating/forming die, converge, driven by the tapered geometry of the cavity and are compacted and formed to the designed cross-section shape. The cooling system absorbs the heat from the processed material, determining the solidification of the thermoplastic matrix and the consolidation of pultruded profile. The geometry of the cavity gives the macroscopic shape to the final composite. However, evident irregularities are visible on the external surface of the pultruded, as observable in Figure 1c. They can be ascribed to the low number of tapes used, the loss of a small fraction of the matrix during the process, and the thermal shrinkage during the cooling. The last factor also influenced the material–die interaction.

**Figure 4:**
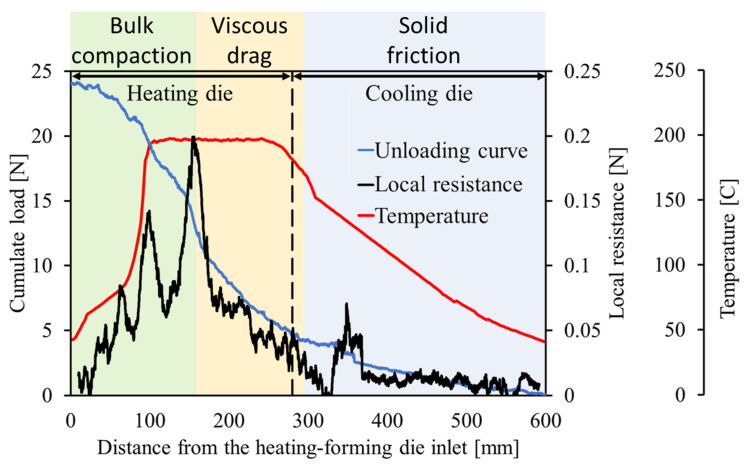
Unloading, local resistance, and core temperature within the dies.

In steady-state conditions, the pulling force necessary to move the materials and process the tapes was 24 ± 2 N. The load measurements conducted during the unloading stage highlight the local contribution to the resistance to the pulling action. In Figure 4, the unloading curve and the local resistance curve computed by using Equation (2) are compared to the profile core temperature measured by the traveling thermocouple method.

The material enters the heating/forming die at room temperature. Initially, the tapes, traveling through the converging section, are not fully compacted. The loose contact between the tapes, and between the advancing material and the die walls, determines a low transversal heat flow and reduces the local resistance of the material to the pulling. The core temperature, indeed, slightly increases throughout the earliest portion of the cavity. These effects are visible until 80 mm from the die inlet, where the temperature sharply increases. Until this point, the local resistance is irregular, with several peaks and an oscillating pattern due to the discontinuous contact between material and cavity walls. Nevertheless, the average local resistance increases due to the progressive compaction action exerted by the convergent cavity. The higher compaction of the pultruded materials is the main factor responsible for the local resistance increase. The effects of compaction are visible also in the unloading curve. Indeed, between 80 mm and 160 mm from the heating die inlet, the slope of the curve appears remarkably higher. The better compaction occurring in the second half of the tapered cavity also improves the heat transfer from the die walls to the core of the material, determining a sharp change in the temperature curve slope. The temperature overcomes the melting point of the polypropylene matrix and stabilizes at about 200 °C.

Starting from 100 mm from the inlet, the matrix starts melting, provoking a lubrication action and causing a sharp decrease in the local resistance. On the other hand, once the polypropylene completely melts, it behaves as an incompressible liquid, determining an increase in pressure and, as a consequence, a peak in the resistance. Even if the higher load should be generally avoided to limit residual tension, the increase in the local pressure in the converging cavity is beneficial to the promotion of transversal matrix flow and, thus, to better inter-tape bonding. The peak value of the local resistance observed at the end of the converging cavity is the maximum value detected throughout the two dies.

Afterward, the die has a straight cavity, where the viscous drag is the unique active force. The temperature remains constant up to 250 mm from the heating die inlet. After that point, the material starts cooling and continues throughout the cooling die. The temperature decreases and the matrix solidifies. A minor resistance peak was detected 350 mm from the inlet. Here, the thermal conditions cause the liquid–gel–solid transition, which increases the local viscosity of the matrix and determines an adhesive contact between the processed materials and the die walls. Within the final 250 mm of the cooling die, the unloading curve presents a linear decrease with a constant mild slope. Here, the interaction between the die and the compacted composite profile is based on the solid friction, which is remarkably lower than the force peaks observed in the converging region.

**Figure 5:**
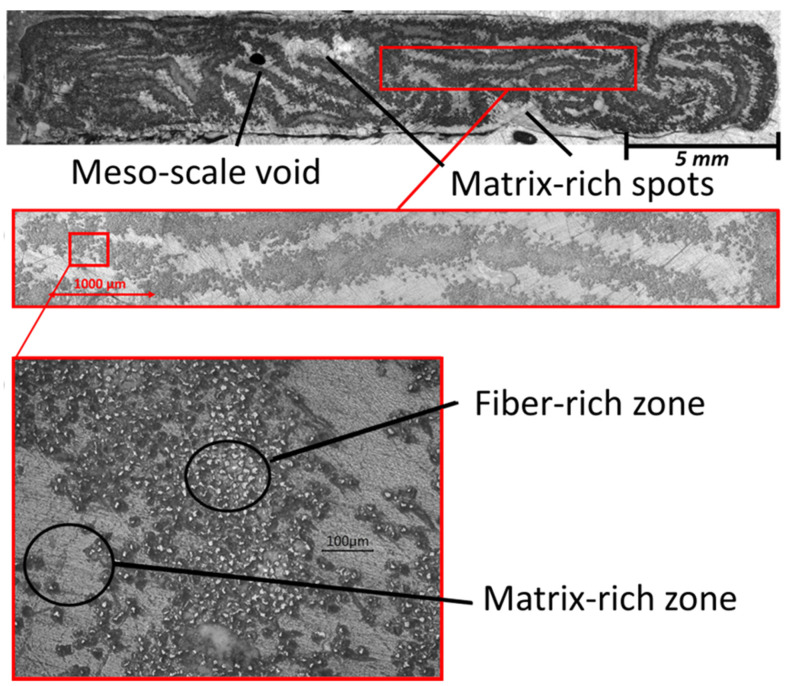
Macrography and optical microscopy of the cross-section of pultruded profile at different magnifications.

The macrograph and micrographs of the cross-section of the pultruded profile are reported in Figure 5. The images point out that the uneven external surface observed can be ascribed to the fibrous architecture and how the tapes are distributed within the profile.

The pultruded profile presents a satisfactory overall distribution of the glass fibers covering almost the entire cross-section. However, the fibers did not appear to be uniformly distributed in the cross-section, and the remained mostly grouped in the original tapes as they entered the die. This is clearly visible in Figure 5b. This reinforcement grouping determines the presence of large resin-rich zones, in which the absence of fibers may lead to lower mechanical strength. In these weak zones, eventual cracks, indeed, could easily propagate and determine the catastrophic failure of the profile during its in-service life. Despite this aspect, the distribution of the reinforcement in the pultruded profile obtained from the commercial pre-consolidated tapes can be considered acceptable and is superior in quality with respect to composites from towpregs and commingled yarns. The pultruded composite presented the fiber as almost completely wetted by the resin and without evident dry spots [23]. Novo et al. [23] suggested that the reason must be ascribed to the way the pre-impregnated raw materials are produced and the state of their impregnation. Commingled yarn and pre-impregnated tapes present fibers that are well impregnated before the processing, with resin and fibers uniformly distributed and in intimate contact, while the two-pregs are likely to have an uneven distribution of the polymeric particle, which causes areas rich in fibers or in matrix, or very large dry spots [23]. Furthermore, one meso-void having a main dimension of 0.6 mm is visible in the core of the profile. The void appears strictly surrounded by the fibrous gathering relative to three tapes. This can be ascribed to the lack of matrix flow, probably related to the high viscosity of the melted thermoplastic, and the configuration of the compacted tapes. It is worth noting that intra-bundle voids were not detected inside the pultruded profile (see Figure 5c), differently from what was observed by Vedernikov and Novo [16,23], who found elevated percentages of voids and dry spots in composites pultruded from pre-consolidated tapes and the other pre-impregnated materials. This confirms that both the production of the raw materials and the optimization of the pultrusion process conditions play key roles in the quality of the final piece.

**Figure 6:**
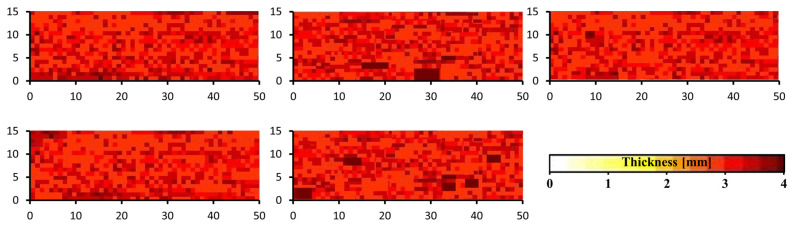
Mapped scans of the windows investigated by ultrasonic inspection on 15 × 50 mm wide scanning windows.

The results of the ultrasonic inspections performed on five random investigation windows of the pultruded profile are reported in Figure 6, which shows the C-scan graphs.

The scans highlight that in the investigated sectors, the depth of the main reflection ranges between 3.7 mm and 4 mm, pointing out that no macroscopic process-related internal defects have been detected inside the profile. It is also evident that the thickness of the composite profile is not homogeneous. The uneven surfaces detected by the visual inspection and the microscope observations are the reason for the inhomogeneity in the reflection depth and cause the variability in the mapped scans.

**Figure 7:**
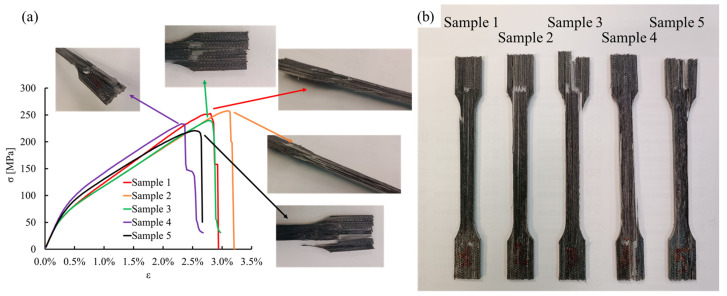
(**a**) Stress–strain curves and close-up of the samples’ failure; (**b**) failed samples.

The microstructure of the pultruded composite directly affects the mechanical behavior and the failure mechanisms under tensile and bending conditions. The stress–strain curves resulting from the tensile testing are reported in Figure 7a.

The five samples exhibited linear behaviors with a tensile modulus of 22.5 ± 0.5 GPa for strain values lower than 0.25%. The ultimate load achieved by the samples is strictly related to the failure mode. Samples 3, 4, and 5 failed at the grip section due to the reinforcement discontinuity generated by the cutting operation, as is observable in Figure 7b, and may be not representative of the actual mechanical behavior of the composite material. Samples 1 and 2 exhibited higher tensile strength of 253 MPa and 258 MPa, respectively. In these cases, the prevalent failure mechanism observed was interlaminar sliding.

**Figure 8:**
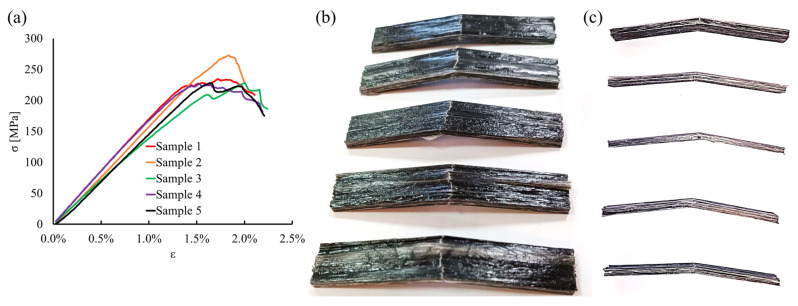
(**a**) Three-point bending stress–strain curves; top view (**b**) and side view (**c**) of the failed samples.

The flexural behavior is represented by the stress–strain curves from the three-point bending test in Figure 8a.

The curve evidences an initial linear response with a flexural stiffness of 15.6 ± 1.5 GPa. During the flexural testing, linear behavior was observed at least up to a strain of 1.3%. The failure points range between 220 MPa and 270 MPa. Figure 8b,c shows the top and side views of the samples after three-point bending testing. The fracture appears neat and transversal. The samples do not evidence the occurrence of delamination nor interlaminar shear, demonstrating good compaction of the tapes. The mechanical properties of the pultruded thermoplastic composites here analyzed are consistent with the data available in the literature, as reported in Table 1. The lower values of flexural and tensile modulus have to be surely ascribed to the reduced fraction of reinforcement obtained in the present investigation. However, the normalized properties with respect to the fiber volume fraction are close to those claimed by other authors. The inferior flexural strength observed here resulted from the presence of larger resin-rich areas on the surface and inside the profiles, and from the imperfect distribution of the fibers, which remained almost gathered in the pristine tapes. These factors contributed to decreasing the flexural properties. Nevertheless, the pultruded composite exhibits notable mechanical behavior in terms of longitudinal elastic modulus. The difference, in this case, should be related to the uncertainty derived by calculating the volume fraction by image analysis, which may be lower than the actual values of the samples investigated here [24,25,26,27,28]. Furthermore, both tensile and bending tests pointed out a high degree of ductility of this material and a good response to high deformation, which could be intriguing for many applications.

The mechanical testing on the produced pultruded composites revealed that, despite the apparent irregularities in the products, their properties are comparable with the conventional materials and, thus, compatible with the engineering applications, and the final quality of the composites is higher than those produced with the other pre-impregnated materials, especially if compared with towpregs thanks to the higher quality of the pre-impregnation [16,23]. Nevertheless, the process needs to be further tuned and optimized in order to achieve higher-volume fractions and a better distribution of the fiber inside the pultruded profile.

## 4. Conclusions

The pultrusion of pre-impregnated tapes in glass-reinforced polypropylene was investigated in the present work, which highlights the potentialities and the challenges related to this process. From the combined study of process forces, microstructure, and mechanical properties, some conclusions can be drawn.

1. Using a pulling speed of 170 mm/min and a heating temperature of 200 °C to pultrude a rectangular cross-section profile having a dimension of 25 × 4 mm and fiber volume fraction of 23%, the pulling force amounted to about 24 N.
2. The analysis of the local resistance and core temperature evidenced that the most critical zone is the final portion of the converging cavity of the heating/forming die. Here, the melted resin is forced towards a narrower cavity, provoking a marked rise in the internal pressure.
3. The fibrous reinforcement is not uniformly distributed over the cross-section of the profile and appears grouped in the configuration of the pristine tapes. Optimization of the pulling speed and processing temperature can potentially improve the polymer flow and be beneficial for a better distribution of the reinforcement.
4. The ultrasonic scan did not detect macroscopic voids or internal defects but confirmed the marked irregularity of the external surfaces.
5. Tensile modulus of 22.5 GPa and flexural modulus of 15.6 GPa were measured. Tensile strength and flexural strength of 255.5 MPa and 245 MPa, respectively, were measured. The normalized tensile and flexural properties are consistent with pultruded thermoplastic composites obtained from pre-impregnated materials.

Further studies should be conducted to analyze the tape pultrusion process and the mechanical performances of the composites pultruded using different processing parameters.

## Figures and Tables

**Table 1:** Mechanical properties of GF/PP pultruded thermoplastic composites.

	PTCs	[23]	[16]	[16]	[29]
Fiber volume fraction	0.23	0.3	0.36	0.32	0.35
Flexural modulus (GPa)	15.6 ± 1.5	16.8 ± 1.5	26.6 ± 0.8	25.7 ± 2.9	23 ± 0.45
Flexural modulus/volume fraction (GPa)	62.4 ± 6.0	56.0 ± 5.0	73.9 ± 2.1	80.3 ± 9.0	65.7 ± 1.3
Flexural strength (MPa)	245 ± 25	329.0 ± 30	485 ± 58	235 ± 91	465 ± 24
Flexural strength/volume fraction (MPa)	980.0 ± 100	1096.7 ± 100	1347 ± 160	734 ± 285	1329 ± 69
Tensile modulus (GPa)	22.5 ± 0.5	21.4 ± 1.5	24.9 ± 1.4	25.2 ± 0.7	-
Tensile modulus/volume fraction (GPa)	90.0 ± 2.0	71.3 ± 5.0	69.2 ± 3.9	78.8 ± 2.2	-
Tensile strength (MPa)	255.5 ± 2.5	355.8 ± 53.2	597 ± 55	561 ± 35	-
Tensile strength/volume fraction (MPa)	1022 ± 10	1186.0 ± 177.3	1658 ± 153	1753 ± 109	-

## Data Availability

The data presented in this study are available on request from the corresponding author. The data are not publicly available due to privacy reasons.
